# Surgical prioritization based on decision model outcomes is not sensitive to differences between the health-related quality of life values estimates of physicians and citizens

**DOI:** 10.1007/s11136-023-03544-5

**Published:** 2023-11-08

**Authors:** Anouk M. I. A. van Alphen, Eline M. Krijkamp, Benjamin Y. Gravesteijn, Robert J. Baatenburg de Jong, Jan J. Busschbach

**Affiliations:** 1 Department of Otorhinolaryngology, Erasmus University Medical Center, Rotterdam, The Netherlands; 2 Department of Epidemiology, Erasmus University Medical Center, Rotterdam, The Netherlands; 3 Present Address: Erasmus School of Health Policy and Management, Erasmus University, Rotterdam, The Netherlands; 4 Department of Obstetrics and Gynaecology, OLVG, Amsterdam, The Netherlands; 5 Department of Public Health, Erasmus University Medical Center, Rotterdam, The Netherlands; 6 Department of Obstetrics and Gynaecology, Amsterdam University Medical Centers, Amsterdam, The Netherlands; 7 Department of Medical Psychology, Erasmus University Medical Center, Rotterdam, The Netherlands

**Keywords:** Medical decision-making, Quality of life, Health care planning, Surgery, Delphi technique

## Abstract

**Purpose:**

Decision models can be used to support allocation of scarce surgical resources. These models incorporate health-related quality of life (HRQoL) values that can be determined using physician panels. The predominant opinion is that one should use values obtained from citizens. We investigated whether physicians give different HRQoL values to citizens and evaluate whether such differences impact decision model outcomes.

**Methods:**

A two-round Delphi study was conducted. Citizens estimated HRQoL of pre- and post-operative health states for ten surgeries using a visual analogue scale. These values were compared using Bland–Altman analysis with HRQoL values previously obtained from physicians. Impact on decision model outcomes was evaluated by calculating the correlation between the rankings of surgeries established using the physicians’ and the citizens’ values.

**Results:**

A total of 71 citizens estimated HRQoL. Citizens’ values on the VAS scale were − 0.07 points (95% CI − 0.12 to − 0.01) lower than the physicians’ values. The correlation between the rankings of surgeries based on citizens’ and physicians’ values was 0.96 (*p* < 0.001).

**Conclusion:**

Physicians put higher values on health states than citizens. However, these differences only result in switches between adjacent entries in the ranking. It would seem that HRQoL values obtained from physicians are adequate to inform decision models during crises.

**Supplementary Information:**

The online version contains supplementary material available at 10.1007/s11136-023-03544-5.

## Introduction

COVID-19 has provided unprecedented shocks to healthcare systems worldwide with consequences that will continue long after the pandemic has subsided. One of the consequences for surgical disciplines was a growing surgical backlog due to the allocation of surgical capacity to intensive care units [1–3]. Future crises may cause similar or even greater difficulties. Therefore, thought needs to be given to the allocation of scarce surgical resources.

The allocation of surgical resources (e.g. operating room capacity) is still largely based on historical patterns or decision-making driven by intuition [4]. In recent years, increasing attention has been given to systematic and evidence-based approaches such as decision analysis to inform resource allocation [5]. In decision analyses, relevant trade-offs are captured in a decision model. These models estimate the outcome of various strategies in the presence of uncertainty.

Health-related quality of life (HRQoL) values are frequently used as input parameters for decision models. HRQoL refers to the health aspects of QoL [6]. Here, the HRQoL gain is seen as a measure of the benefit of a treatment. Nevertheless, there is a continuing debate about methodological design choices used in HRQoL assessment studies [7–10]. Concerns include the methods used in evaluation and whose values should be used. Leading national and international guidelines for cost-effectiveness analysis recommend methods where patients report their own health state, using (EuroQol-5 dimension) EQ-5D, and then normative values, that are based on the views of the general public, are applied [11–13].

Amidst the COVID-19 crisis, we developed a decision model to support the prioritization of surgical care in times of scarcity. The impact of surgical delays on health for elective and semi-elective surgeries was estimated [14, 15]. Notably, in our model, we deviated from the international recommendations for HRQoL estimates, and instead obtained estimates through a physician panel using the visual analogue scale (VAS) method. This was for two reasons. First, the rapidly evolving COVID-19 crisis demanded the rapid development of decision models. Consequently, HRQoL valuations were needed immediately, which was only feasible in the existing situation by using physicians. Second, physicians can be seen as a desirable panel for the determination of HRQoL values as they are skilled and, collectively offer, a broad view and depth of expertise that should enable them to make comparative judgments across a range of diseases.

One could offer the counter-argument that the general public and physicians value health states differently. This could impact decision model outcomes and, subsequently, surgical prioritizations. Therefore, the aims of the current study are two-fold. First, HRQoL values estimated by physicians will be compared with the HRQoL estimates from citizens. Second, the impact of the choice of respondent panel on the decision model outcomes and subsequent ranking of the surgical procedures will be assessed.

## Methods

In our previous studies, physicians had estimated HRQoL values for 43 semi-elective surgeries frequently performed in academic hospitals [14, 16]. For each surgery, the health loss, expressed as disability-adjusted life years (DALY) per month of surgical delay, was calculated. This outcome was subsequently used to compose a ranking of these surgeries. We argue that the surgery with the greatest health loss should be prioritized. A detailed description of this preliminary work can be found in an earlier publication[14]. For the current study, a sample consisting of ten surgical interventions deliberately selected from the 43 surgeries was used. Three inclusion criteria were applied: (1) the sample should be representative of different surgical specialties, (2) oncological and non-oncological diseases should be included, and (3) they should be spread across the earlier ranking of 43 surgeries based on health loss per month of surgical delay (including the extreme ends). An overview of the surgeries included is provided in Table 1.

**Table 1:** Sample of ten surgeries evaluated in the current study

Surgery	
1	Peripheral arterial disease Fontaine 3–4 (PAD F3-4), bypass
2	Renal cancer, total nephrectomy
3	Aortic valve replacement (AVR), transcatheter aortic valve (TAVI)
4	End-stage liver disease (ESLD), transplant
5	Penis cancer, resection
6	Hepatocellular cancer (HCC), resection
7	Endometrium cancer, resection
8	Empyema, video-assisted thoracoscopy (VATS)
9	Severe salivary gland cancer, resection
10	End-stage renal disease (ESRD), shunt

For each surgery, we established a description of the pre- and post-operative health states, the so-called “vignettes”, for use with our citizen panel. These were derived from vignettes used in earlier studies [14, 16]. In live panel sessions, experienced physicians described health states of typical patients, while others asked clarifying questions [14]. These vignettes were documented and reused in the follow-up study after a collective review with our research group [16]. In the current study, these vignettes were again employed. There is no standard regarding the content of such vignettes [17], and so we adhered to the following principles: vignettes should be as brief as possible, written in simple language, and describe the typical patient. They included descriptions of symptoms and effect on activities associated with daily living. These descriptions were similar to those we had used in our previous studies with physicians but without any medical jargon. An overview of the vignettes and VAS is shown in Online Resource 1.

Citizens were recruited through a panel company. To compose a balanced panel that was representative of the Dutch population, the participants were sampled on age, sex, and socioeconomic status. Two independent citizen panels were established (CP1 and CP2) to (1) validate the results obtained using the physician panel, and (2) to compare the results of each citizen panel to each other to establish their reliability. We aimed for 15–20 citizens per panel as this is within the typical range of other Delphi studies [18, 19]. To obtain HRQoL values from physicians, we used data from our previously published studies [16]. This physician panel was purposefully chosen because they had previously valued the same health states using the same method, which will be discussed below.

### HRQoL data collection

A two-round Delphi study was conducted to collect HRQoL data from citizens in January 2022. Due to COVID-19 restrictions, an online web-based Delphi method tool named ‘Welphi’ was used [20]. Citizens received an invitation from the panel company to participate in the current study. This invitation contained a short description on the expected time investment and study procedures. No background information about the study was presented. Members of the panel who were interested in participating in our study were redirected to Welphi by clicking on a link in the invitation.

HRQoL data were collected in accordance with the valuation method described by Stouthard et al. through which health states are rated [21]. The structure of the HRQoL valuation was as follows. In the first round, each citizen was presented with two vignettes describing the pre-operative and post-operative health states for each of the ten surgical interventions. Citizens were asked to estimate the HRQoL value of each health state using a calibrated visual analogue scale (VAS). This scale is a measure ranging from 0 (“worst imaginable health”) to 100 (“best imaginable health”) [22]. Five available HRQoL estimates (for dementia, severe depression, blindness, deafness, and infertility) from the World Health Organization Global Burden of Disease study were made available to provide reference points [23]. Citizens were instructed to indicate the position of each given health state on the scale by giving a number between 0 and 100. Next, citizens were asked to give a short comment on their HRQoL estimate.

Two weeks after completion of this first round, the citizens received an email invitation for the second round. In this round, citizens were presented with the same vignettes and tasks. However, in this second round, a selection of the comments, the median HRQoL value, and the interquartile range from the first round of the their panel were displayed. Citizens were then able to alter their HRQoL estimate given in the first round. After completion of both rounds, some citizens’ responses were excluded from the analyses if their answers seemed doubtful. Here, the following exclusion criteria were applied: (1) negative HRQoL estimates, (2) only HRQoL estimates equal to zero, (3) consistently lower HRQoLs in the post-operative health state.

### Decision model

The ranking of surgical procedures is established by the decision model outcomes. We used our previously developed decision model to estimate the health effects of delay for these ten interventions using citizens’ HRQoL values [14]. The model is a three-state cohort state-transition model which requires seven input parameters: (1) survival rate pre-surgery, (2) survival rate post-surgery, (3) HRQoL pre-surgery, (4) HRQoL post-surgery, (5) mean age of patient undergoing the surgery, (6) time until no effect of treatment can be expected on survival, or (7) time until no effect of treatment can be expected on HRQoL. The three health states considered in this model were pre-operative, post-operative, and deceased. The entire cohort started in the pre-operative state, followed by a transition to the post-operative or deceased state. The scenarios modelling surgical delay were created with intervals of ten weeks, starting from two weeks up to one year. Permanent cancellation of surgery was modelled as patients remaining in the pre-operative health state until they died. The cohort was simulated over a lifetime which was defined as lasting until they became 100 years old. The model outcome used in the current study to evaluate the impact of HRQoL values is DALY per month of surgical delay. In line with the utilitarian ethical perspective, priority should be given to patients with the highest DALYs per month of surgical delay. A detailed description of the decision model and our rationale for prioritization can be found in our previous work [14].

### Analysis

A Bland–Altman analysis was used to evaluate the agreement between (1) the two citizen panels (CP1 and CP2), and (2) all citizens (CP) and physicians (PP). For each analysis, the pre- and post-operative HRQoL values were analysed separately. Recommendations for reporting a Bland–Altman analysis were applied in our study [24]. First, lower and upper levels of agreement were calculated and the normal distribution of the mean differences was visually inspected. Further, the mean difference (i.e. estimated bias) was determined by fitting a linear mixed effects model with a random intercept for each of the surgical procedures and each participant. HRQoL was the dependent variable, and the independent variables were panel and health state (pre-operative and post-operative).The general criterion is that if the 95% confidence interval of the mean difference includes zero then there is no significant bias. Clinically acceptable limits of agreement could not be established a priori since, to the best of our knowledge, there is no consensus on the minimum clinically significant difference in HRQoL values established using VAS. Standard deviations were compared between the citizen panels, and between all citizens and physicians to explore the degree of consensus among the respondent panels. A lower standard deviation represents a higher degree of consensus as there is less variability.

To explore whether using HRQoL values determined by citizens impacted on the decision model outcome, we used these in place of the HRQoL model input parameter values estimated by physicians in our previously published model. The other input parameters (e.g. survival, age) were kept the same as the values used in the previously obtained data [14]. All parameter values were sampled from their distribution in the probabilistic sensitivity analyses. The impact on model outcomes was evaluated by calculating the Spearman’s rank correlation between the two rankings established using either the physicians’ or the citizens’ values. Analyses were conducted using R open source software [25]. The lmer function in the lme4 package was used to establish the linear mixed effects model [26].

The Medical Research Ethics Committee of Erasmus University Medical Center reviewed the proposed research and waived the requirement for ethical approval for this study (reference number: MEC-2021-0612). This study had no external funding source. The Strengthening the Reporting of Observational Studies in Epidemiology (STROBE) statement was used to guide the study’s reporting (Online Resource 2) [27].

## Results

### HRQoL valuation

A total of 720 citizens were invited to participate by the panel company. First, all these citizens (CP) were divided into two equal groups of 360 (CP1 and CP2). With CP1, the number of completed Welphi responses in the first round was 47, and all these were invited for the second round. After completing the second round, 8 citizens were excluded due to unacceptable answers (negative HRQoL estimates (*n* = 1), HRQoL estimates equal to zero (*n* = 1), and lower estimates for post-operative state (*n* = 6)). This resulted in the final CP1 consisting of 39 citizens. With CP2, 41 completed the first round of whom 9 were excluded in the second round (negative HRQoL estimates (*n* = 2), HRQoL estimates equal to zero (*n* = 4), and lower post-operative estimates (*n* = 3)). This led to a total of 32 citizens in CP2. A flowchart of the study procedure and mean HRQoL values obtained is shown in Online Resource 2. In the previous study evaluating physicians’ HRQoL valuation, 15 physicians participated (the PP), 8 of whom were from surgical specialties. Detailed information regarding the composition of this panel can be found in our earlier publication [16].

### Bland–Altman analysis

**Fig. 1:**
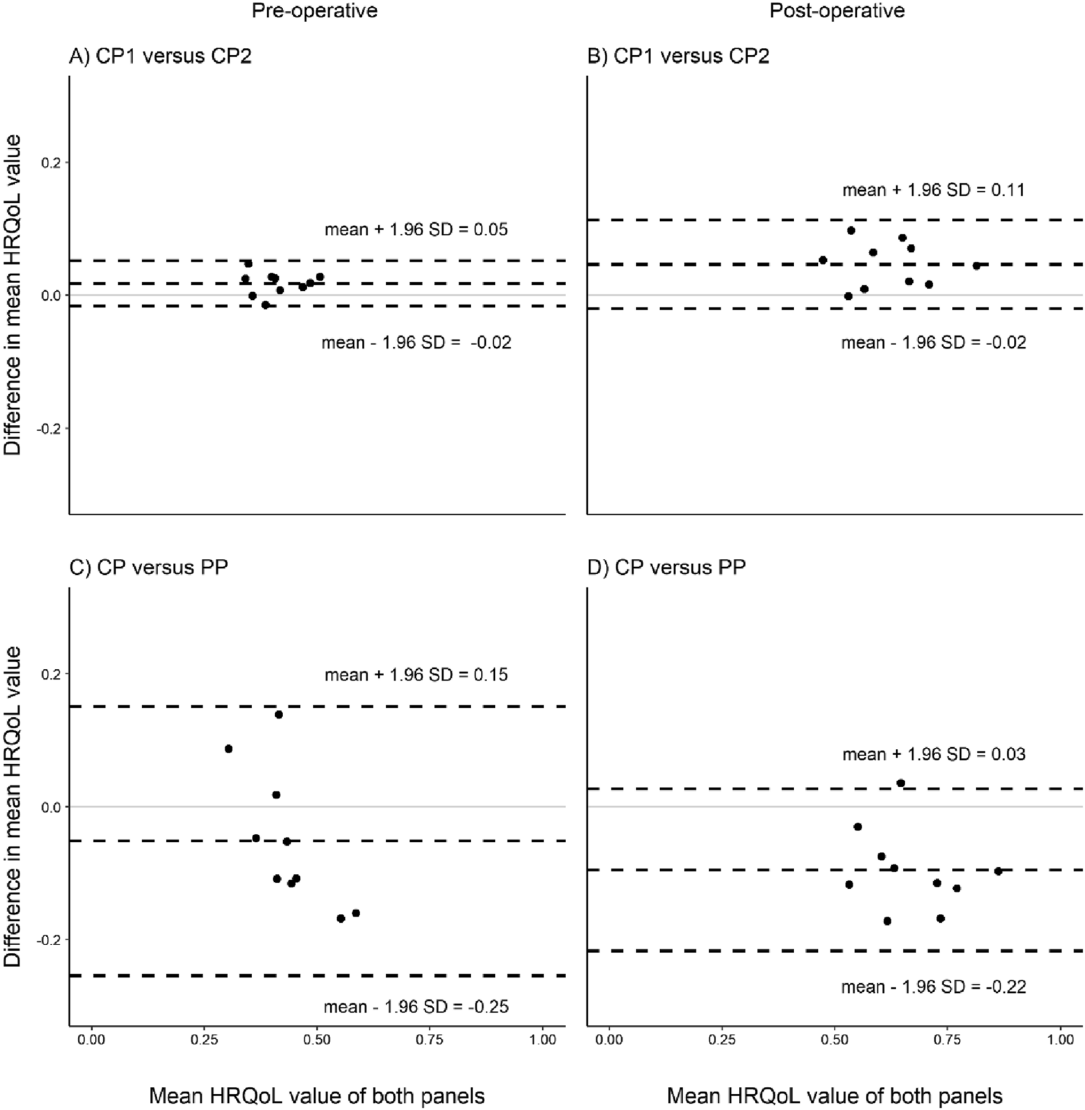
Bland–Altman plots of the HRQoL values from CP1 and CP2 (upper) and all citizens (CP) and physicians (PP) (lower). All plots are stratified for the pre-operative (left) and post-operative (right) health states. Mean *A* = 0.02, mean *B* = 0.05, mean *C* = − 0.05, and mean *D* = − 0.10. The *y*-axis shows the mean difference in HRQoL values and the *x*-axis represents the average of the HRQoL values based on data from two respondent panels. Each dot represents a vignette. The dashed horizontal lines represent the Bland–Altman bias and 95% limits of agreement

The mean difference between all the HRQoL values of the two citizen panels was 0.03 (95% CI − 0.02 to 0.08). After making subgroups of the HRQoL values for the pre-operative and post-operative health states, the Bland–Altman mean difference and lower and upper levels of agreement were 0.02 (95% CI − 0.02 to 0.05) for the pre-operative and 0.05 (95% CI − 0.02 to 0.11) for the post-operative health states. In general terms, the mean difference can be interpreted as the estimated bias. As such, CP2 is expected to have a structurally lower HRQoL value of around 0.03 points. The difference is 0.02 when only looking at the pre-operative health states, and 0.05 when looking at the post-operative health states. Histograms of these differences suggest a roughly normal distribution. The upper part of Fig. 1 shows the results for the Bland–Altman analysis for CP1 versus CP2.

The mean difference of HRQoL values between physicians (PP) and all citizens (CP, *n* = 71) was -0.07 (95% CI − 0.12 to − 0.01). For the pre-operative health states, the Bland–Altman mean difference and lower and upper levels of agreement were − 0.05 (95% CI − 0.25 to 0.15). For the post-operative health states, the equivalent values were − 0.10 (95% CI − 0.22 to 0.03). The lower part of Fig. 1 illustrates the results of the Bland–Altman analysis comparing the physician panel with all citizens.

**Fig. 2:**
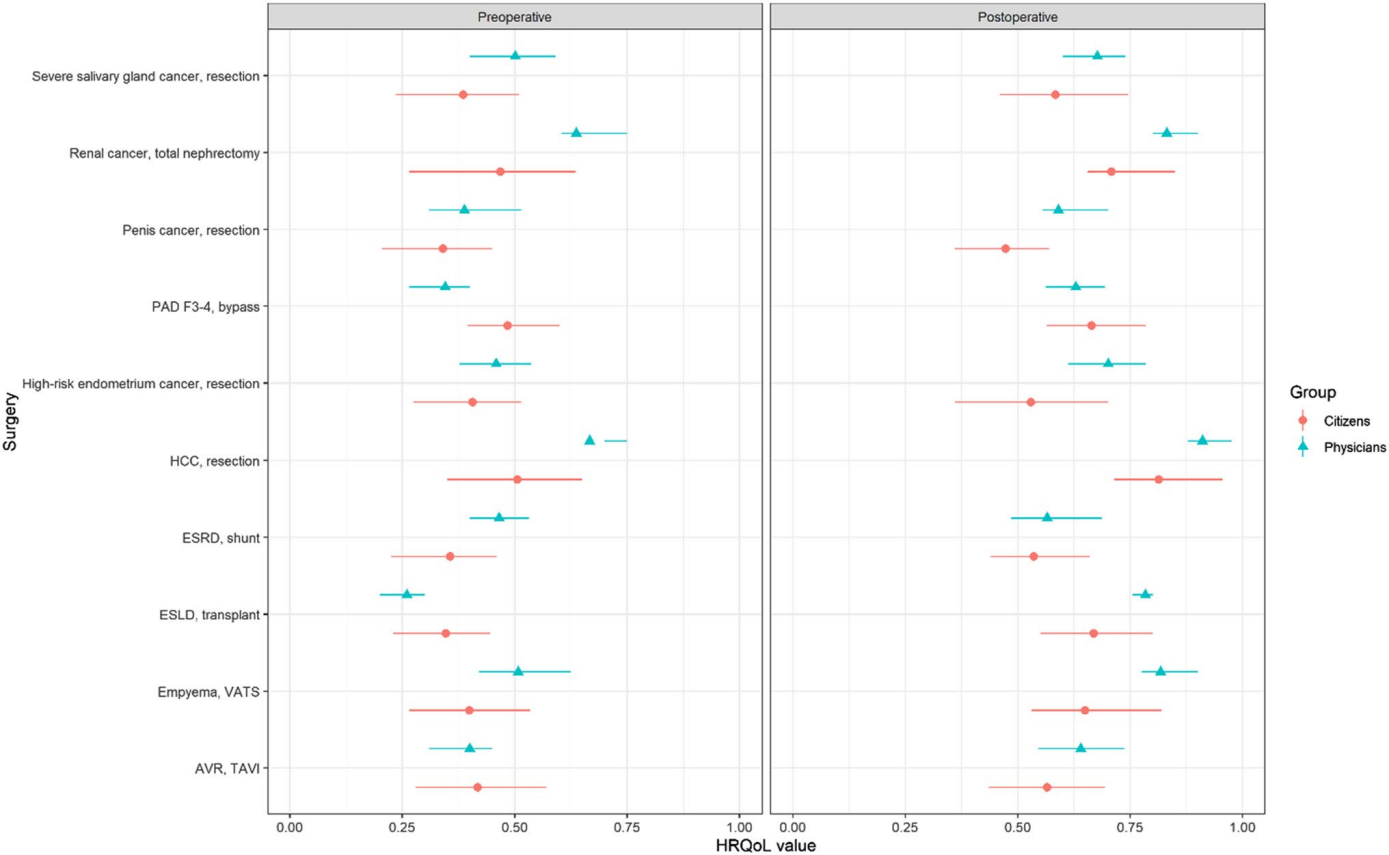
HRQoL values estimated by the citizens and physicians, stratified for the pre-operative (left) and post-operative (right) health state. *AVR* aortic valve replacement, *ESLD* end-stage liver disease, *ESRD* end-stage renal disease, *HCC* hepatocellular cancer, *PAD F3-4* peripheral arterial disease Fontaine 3–4, *TAVI* transcatheter aortic valve, *VATS* video-assisted thoracoscopy

The standard deviations of the HRQoL values were consistently higher for the citizens compared with those of the physicians (standardized mean difference of 0.07 (95% CI 0.09 to 0.05). As such, we concluded that the degree of consensus among citizens (*n* = 71) was systematically lower than with the physicians. However, when comparing both citizen panels with each other, a high degree of consensus between the two was observed, with a standardized mean difference of only 0.01 (95% CI 0.00 – 0.03). An overview of all mean values and standard deviations is shown in Fig. 2 and Online Resource 2.

### Ranking

**Fig. 3:**
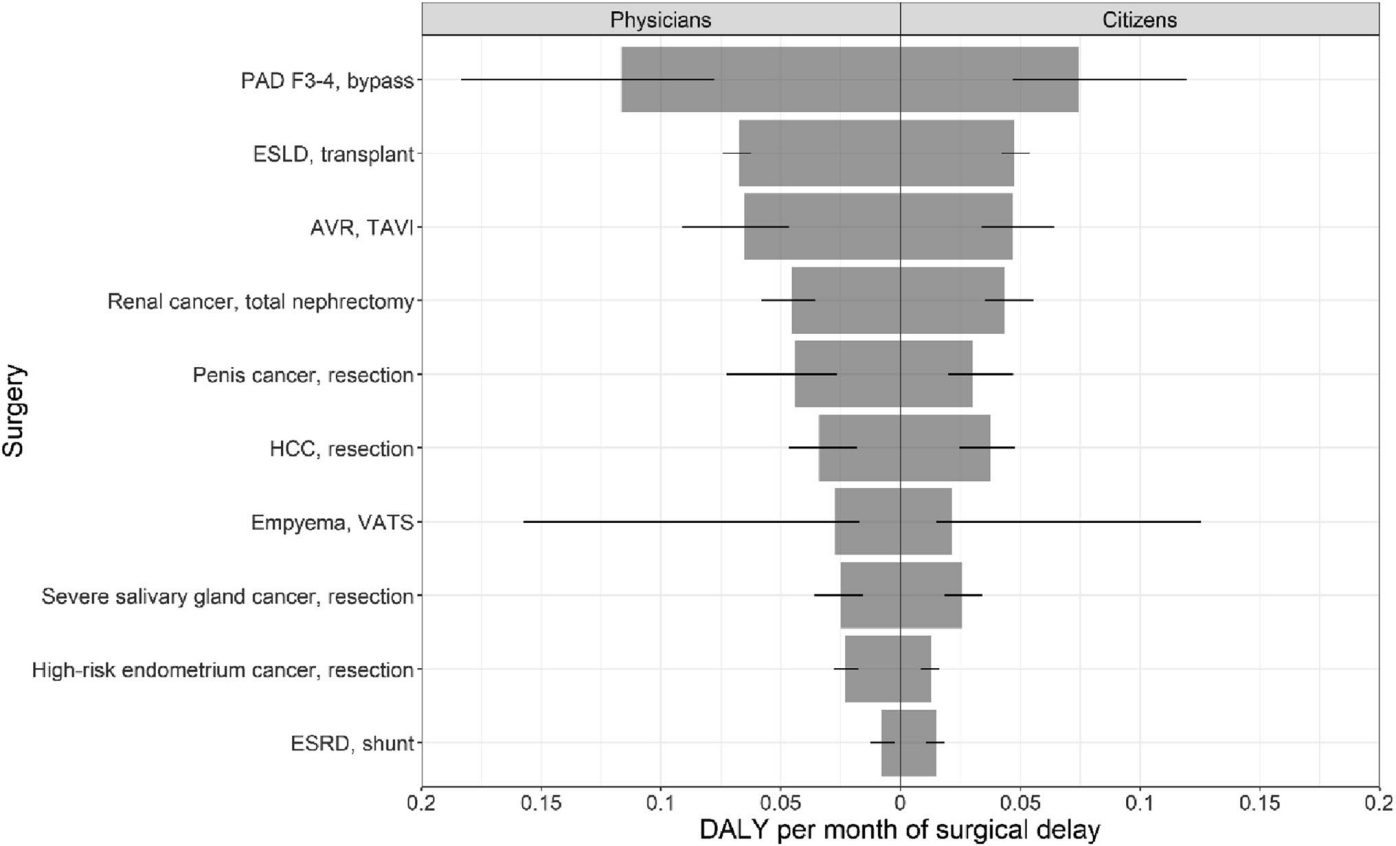
Model outcome established using HRQoL values from physicians (left) and all citizens (right). The average DALYs per month of surgical delay are displayed. The estimates (grey bars) and 95% confidence intervals (black lines) are shown. *AVR* aortic valve replacement, *ESLD* end-stage liver disease, *ESRD* end-stage renal disease, *HCC* hepatocellular cancer, *PAD F3-4* peripheral arterial disease Fontaine 3–4, *TAVI* transcatheter aortic valve, *VATS* video-assisted thoracoscopy

The model outcome (i.e. DALY per month of surgical delay) stratified for respondent panel (i.e. citizens or physicians) is shown in Fig. 3. Bypass surgery for the peripheral arterial disease Fontaine, stage 3–4, was associated with the most DALY per month of surgical delay by both respondent panels. The least DALY per month of surgical delay based on the physicians’ HRQoL values was found for shunt surgery for end-stage renal disease. Conversely, using citizens’ HRQoL values, resection for high-risk endometrium cancer had the least DALY per month of surgical delay. Figure 3 presents the model outcomes using the physicians’ (left) and citizens’ (right) HRQoL values. The thin black lines indicate the 95% confidence intervals and the generally longer lines on the left-hand side reflect the greater variance in the physicians’ HRQoL estimates.

**Fig. 4:**
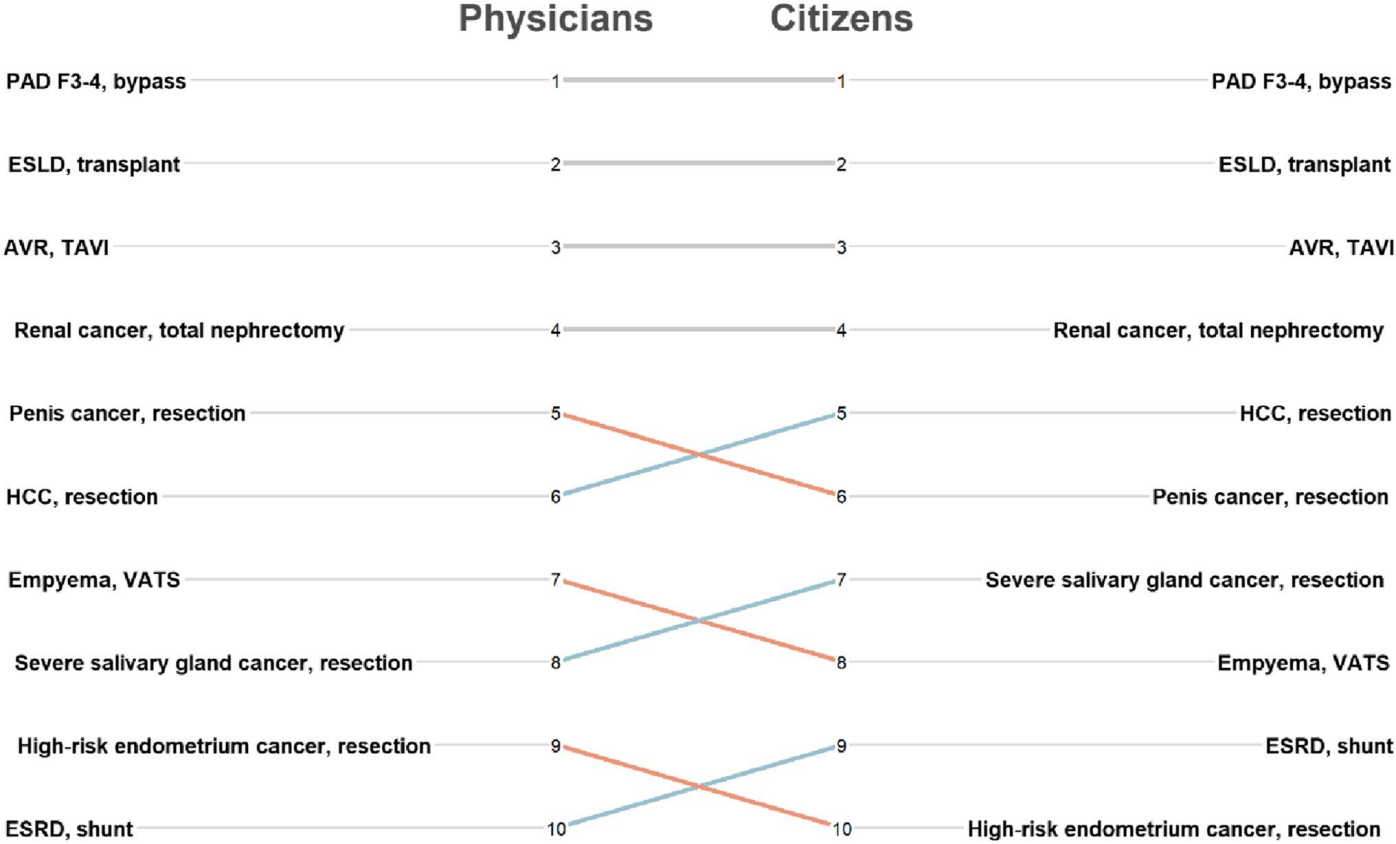
Ranking of surgeries established using HRQoL values from physicians (left) and all citizens (right). The grey horizontal lines represent no shift in ranking position due to using citizens’ HRQoL values compared with physicians’ HRQoL values. The orange lines represent a drop in position, whereas the blue lines show an increase. *AVR* aortic valve replacement, *ESLD* end-stage liver disease, *ESRD* end-stage renal disease, *HCC* hepatocellular cancer, *PAD F3-4* peripheral arterial disease Fontaine 3–4, *TAVI* transcatheter aortic valve, *VATS* video-assisted thoracoscopy

There were only minor differences in the ranking of surgical procedures as established by the model outcomes based on whether we used the values indicated by the physician (PP) or by the citizen (CP) panels. As can be seen in Fig. 4, only the rankings of adjacent entries switched. Indeed, the Spearman’s correlation coefficient (rho) between the ranking based on physicians’ values and the ranking based on citizens’ values was 0.964 (*p* < 0.001).

The largest difference in model outcome was found for the peripheral arterial disease Fontaine stage 3–4. Using physicians’ HRQoL values, the DALY per month was 0.12 (95% CI 0.08–0.18), whereas using the HRQoL values from the citizens yielded 0.07 (95% CI 0.05–0.12) DALY per month. Nevertheless, this difference did not result in a change in the ranking.

## Discussion

We compared the citizens’ HRQoL estimates of health states with those estimated by physicians in a previously published study. It was found that physicians valued health states systematically higher than citizens. Furthermore, the two citizens’ panels (CP1 and CP2) showed a very high degree of consensus, giving us confidence in the reliability of our results. Moreover, the difference in HRQoL values did not substantially alter the decision model outcomes, and the ranking of the surgical interventions remained consistent, with only adjacent entries in the ranking list changing position.

Previous studies that have evaluated the differences between physicians’ and citizens’ HRQoL valuations have produced inconsistent results. In line with our results, some previous studies have found that physicians in general assign higher HRQoL values to health states than do citizens [28–31]. The impact of demographic or contextual differences on HRQoL valuations has been extensively studied [32; 33]. On the whole, these studies found that the impact of these aspects was minor compared to differences that could arise from methodological choices [32].

Since previous studies have highlighted the impact of the method used for establishing HRQoL valuations, it is imperative that we critically appraise our approach. Two alternatives spring to mind: (1) obtain values from patients, rather than physicians and citizens as used in the present study; or (2) using values based on ‘utility measures’ such as the EQ-5D of patients actually in the health states being considered. The consequences of using such alternative approaches is discussed below.

First, one could expect that patient panels whose members have not experienced the specific health state being considered will give the same results as citizens since both of these panels will provide non-experience-based values [34]. This is in contrast with patients evaluating their own health state, i.e. a state that they are currently in or have previously experienced. There is considerable literature describing adaptation phenomena in that patients attach substantially higher values to a health state they are experiencing than do people who are not in the compromised state [34–36]. At the same time, the values given to health states other than their own seem not to be affected by coping, and are most likely similar to values from other respondent panels who are not experiencing the specific health state (e.g. citizens) [37]. As such, any differences between the HRQoL values of patient and of citizen panels are expected to be small.

Another alternative approach would be to obtain HRQoL values from empirical research involving patients experiencing the relevant health states (e.g. EQ-5D questionnaires). Although this would be in line with the current international recommendations for cost-effectiveness analysis [11–13], this approach would have some notable shortfalls in regard of our decision model. Foremost, it is questionable whether one could collect values for all the health states included in the model in one coherent study. The current mode already incorporates 84 surgical interventions [15], and will be continuously updated and extended, so collecting sufficient EQ-5D data would require a tremendous effort. Another option would be to collect data from observational or routine datasets, or to use existing estimates identified in the literature. However, this approach would raise several concerns. First, it is unlikely that health states evaluated in other studies would be representative of our population of interest (i.e. patients in the Netherlands who are awaiting the surgical interventions that are simulated in our model). Given that most published HRQoL values represent patients in randomized clinical trials that have strict inclusion and exclusion criteria, these values are unlikely to be fully representative of the wider population. Such studies might also be compromised by imprecise evidence, poor response rates, and incomplete follow-up data. For the same reasons, the Global Burden of Disease study also avoided using patient-reported HRQoL data [23]. Second, using data from other studies has the risk that one combines different utility measures. This is a concern since it has been demonstrated that different utility measures give quite different results [38–41], and therefore using multiple measures has been strongly discouraged [42].

Although alternative utility measures methods have been proposed, such as time trade-off and standard gamble, which might, at least theoretically, be preferable for HRQoL evaluation, we deliberately opted for evaluating HRQoLs using the calibrated visual analogue scale (VAS) with reference points as introduced in the Global Burden of Disease study. Given the necessarily practical approach of the current study, we considered this to be a reliable and easy-to-interpret measure that allowed us to contemporaneously estimate HRQoL values for multiple diseases. An additional advantage is that we could then use one single measurement instrument and include consistent overall health states, thereby following existing recommendations [42].

Since we only evaluated ten surgical interventions in the current study, this could impact the generalizability of our results. This small number was deliberately chosen for practical reasons. We intended to provide the participating citizens with a short, manageable task to maximize engagement. We foresaw that an online task which took longer than 30 min would be off-putting for potential participants [43, 44], and potentially reduce the response rate. We therefore chose ten surgical interventions with care to cover the spread of likely impacts of surgical delays, believing that evaluating the associated 20 health states in the Delphi study was realistic within this time limit, and would increase the representativeness of the sample.

The fact that there are no standardized descriptions for the vignettes evaluated is another potential concern since the HRQoL values given by the citizens will be highly dependent on these descriptions. Although our vignettes are not all identical in structure, we addressed this limitation by applying general principles [17] (e.g. they should be brief, avoid medical jargon, reflect typical patient experiences) when formulating them. Further, the vignettes provided to the citizen panels were based on those used earlier with the physician panel albeit with any medical jargon omitted or rephrased.

In general, there seems to be a consensus that the source of HRQoL values should match the perspective and the level of decision-making [45–48]. As such, in the case of individual, patient-level decision-making, it is preferable to use patients’ health states. For macro-level decision-making, where HRQoL values are used to compare population health, panels of medical experts can be used. Indeed, there are many studies that have used valuations provided by medical experts for the same reasons as we did [10]. We would argue, when setting surgical priorities, that using values provided by physicians who have a generic perspective is the most appropriate approach. Furthermore, national guidelines [13] recommend using panels of medical experts when HRQoL values representative of the population of interest are lacking. Here, the Delphi method is broadly seen as an acceptable approach to reach a consensus within such panels.

Our findings affirm our model’s reliability, consistent with previous studies [14–16]. In these studies, we assessed the reliability of each physician panel by comparing the HRQoL values obtained from one physician panel to those obtained from another physician panel. Minor, yet consistent, HRQoL variations among physician panels did not affect surgical rankings. Our approach, using physicians to represent societal values, handles complex health state descriptions effectively. Extending our model to include more surgeries using this methodology seems appropriate in crises.

We would like to stress that other factors which are currently not represented as input parameters in our model may well impact decision model outcomes. Therefore, instead of re-evaluating our HRQoL valuation method, it would be more worthwhile to instead consider incorporating patient characteristics such as comorbidities and age in our model since these characteristics are known to influence pre- and post-operative surgical outcomes. As such, such additions would contribute to a much more refined model outcome. Further, we acknowledge that prioritization is, in practice, a complex decision-making process that involves normative criteria, contextual factors, capacity constraints, and stakeholder interests. Ultimately, the usability of our model’s outcomes in daily practice will be greatly influenced by all of these aspects.

## Conclusion

Minimal differences in HRQoL valuations between citizens and physicians had little impact on decision model outcomes in terms of prioritizing surgeries. Regarding our decision model, values from sources beyond the general public can be used, facilitating quicker model extensions which enhance the usability of our model. It is crucial to emphasize that our method should not replace patient-reported outcome measures in routine care.

### Supplementary Information

Below is the link to the electronic supplementary material.

Supplementary file1 (PDF 432 KB)

Supplementary file2 (PDF 677 KB)
