# Supplementary material for: Surgical prioritization based on decision model outcomes is not sensitive to differences between the health-related quality of life values estimates of physicians and citizens
Source: Qual Life Res. 2023 Nov 8;33(2):529–39. doi: 10.1007/s11136-023-03544-5 (PMC10850033; doi:10.1007/s11136-023-03544-5)
Supplement: Supplementary file 1 — Supplementary file1 (PDF 432 KB) [file 11136_2023_3544_MOESM1_ESM.pdf]

Supplementary file 1

Description of vignettes used in this study.

Peripheral vascular disease, bypass surgery

Pre-operative:

Patients have pain in their feet and lower legs because too little blood is flowing to their legs. This particularly happens at night when patients are asleep, but also when patients walk or sit for lengthy periods. Often, wounds form on the feet due to the poor blood flow, which makes walking even more difficult. The general health of these patients is poor.

Post-operative:

After surgery, patients can walk better. The pain in the legs decreases because more blood can now flow to the legs. The general health of these patients improves a little.

Renal cancer, kidney removal

Pre-operative:

Initially, patients have few complaints. They are not limited in their daily life. This is why the cancer is only identified when there is blood in the urine. The fact that these patients are suffering from cancer does give them anxiety.

Post-operative:

Patients are completely cured after this surgery. Nevertheless, there is a chance that the cancer will come back.

Heart valve calcification, replacement of valve

Pre-operative:

Patients are often elderly people in poor health, suffering from multiple illnesses. They suffer from chest pains during exercise and can become short of breath. In addition, they are often tired.

Post-operative:

In general, their health is improved. The patients are still left with the other illnesses, which makes them vulnerable.

Liver failure, replacement of liver

Pre-operative:

Patients feel sick, weak, and nauseous. They are tired all the time and in a bad condition. This surgery is the only chance of a cure.

Post-operative:

Patients generally recover well from this surgery. They no longer feel sick. However, they will have to take medicines that have side effects for the rest of their lives.

Penis cancer, (partial) penile removal

Pre-operative:

Men with penile cancer often have a large wound on the penis. They suffer a lot from this. The fact that these men are suffering from cancer also causes a lot of anxiety.

Post-operative:
Depending on how far the cancer has grown, parts or all of the penis will be removed. After the surgery the complaint and the pain are gone. They can still urinate. However, there is a chance that the cancer will come back. Also, men often feel deformed by the procedure.

Liver cancer, removal of cancer and surrounding tissues in the liver
Pre-operative:
Patients often have few complaints and generally feel good. Patients suffer because they have to live with the thought that they have cancer.

Post-operative:
Patients recover well and are cured after the surgery. The cancer will not come back.

Uterine cancer, removal of uterus
Pre-operative:
Patients are often older women (post-menopause). They have a lot of pain and suffer from bleeding from the vagina. The fact that these women are suffering from cancer also causes a lot of anxiety.

Post-operative:
The uterus is removed during surgery. In addition, patients often have to receive additional chemotherapy. There is a high probability that the cancer will come back or has already spread to other parts in the body.

Pockets of pus in the lungs, removal of pus using keyhole surgery
Pre-operative:
Patients have an accumulation of pus in their chest due to pneumonia. They are often very sick and have a fever. The pus will have been drained several times through a tube, but unfortunately the pus is not completely gone. If the pus remains for a long time, it will become more difficult for the patient to breathe.

Post-operative:
The sickness and the fever are gone. Patients are cured after this surgery, the pus is gone, the tube is no longer needed. Some patients do continue to have difficulty breathing after this.

Salivary gland cancer, removal of salivary gland
Pre-operative:
Patients often have a swelling in the neck, which people find ugly. If the cancer continues to grow, open wounds around the tumor may develop on the skin. The facial nerve can also be affected. This nerve controls the muscles responsible for facial expressions.

Post-operative:

After the surgery, patients always receive radiation at the site where the cancer was located. If the facial nerve is damaged, it cannot be repaired and the muscles in the face are then paralyzed. Fortunately, these complications are not common.

<u>Kidney failure, surgery to create an access point for dialysis</u>

Pre-operative:

In these patients, waste products in the blood have to be removed because the kidneys can no longer do this. This is done through a plastic tube that is inserted into a large blood vessel in the neck or groin. An infection can easily develop around this tube. The process is intensive and painful for the patient. The general health of such kidney patients is low.

Post-operative:

After this surgery, patients no longer need a plastic tube in the neck or groin to have their blood cleaned: the blood now flows through a 'shunt' on the arm. This reduces the risk of infection. However, the general health of these kidney patients remains low.

Figure 1. Visual analogue scale (VAS) used to collect the HRQoL data.

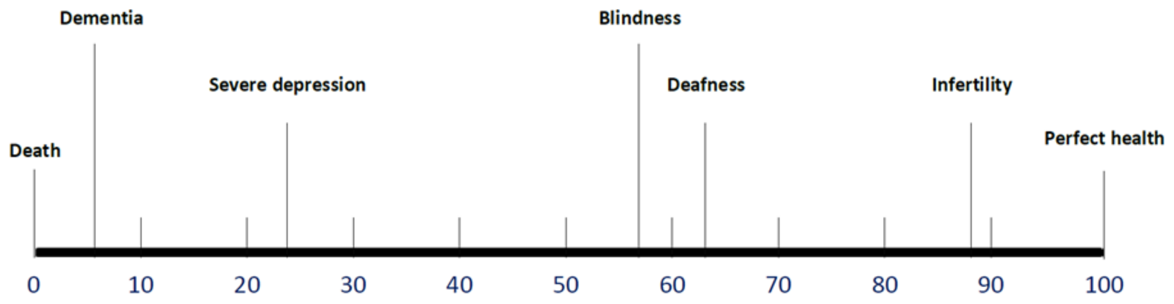

Five available HRQoL estimates (for dementia, severe depression, blindness, deafness, and infertility) from the World Health Organization Global Burden of Disease study were made available to provide reference points.
